# Novel Compound Heterozygous *TMC1* Mutations Associated with Autosomal Recessive Hearing Loss in a Chinese Family

**DOI:** 10.1371/journal.pone.0063026

**Published:** 2013-05-14

**Authors:** Xue Gao, Yu Su, Li-Ping Guan, Yong-Yi Yuan, Sha-Sha Huang, Yu Lu, Guo-Jian Wang, Ming-Yu Han, Fei Yu, Yue-Shuai Song, Qing-Yan Zhu, Jing Wu, Pu Dai

**Affiliations:** 1 Department of Otorhinolaryngology, Head and Neck Surgery, PLA General Hospital, Beijing, P. R. China; 2 Department of Otorhinolaryngology, Hainan Branch of PLA General Hospital, Sanya, P. R. China; 3 Department of Otorhinolaryngology, the Second Artillery General Hospital, Beijing, P. R. China; 4 BGI-Shenzhen, Beishan Industrial Zone, Yantian District, Shenzhen, P. R. China; 5 BGI-Tianjin, Tianjin, P. R. China; Innsbruck Medical University, Austria

## Abstract

Hereditary nonsyndromic hearing loss is highly heterogeneous and most patients with a presumed genetic etiology lack a specific diagnosis. It has been estimated that several hundred genes may be associated with this sensory deficit in humans. Here, we identified compound heterozygous mutations in the *TMC1* gene as the cause of recessively inherited sensorineural hearing loss by using whole-exome sequencing in a family with two deaf siblings. Sanger sequencing confirmed that both siblings inherited a missense mutation, c.589G>A p.G197R (maternal allele), and a nonsense mutation, c.1171C>T p.Q391X (paternal allele), in *TMC1*. We also used DNA from 50 Chinese familial patients with ARNSHL and 208 ethnicity-matched negative samples to perform extended variants analysis. Both variants co-segregated in family 1953, which had the hearing loss phenotype, but were absent in 50 patients and 208 ethnicity-matched controls. Therefore, we concluded that the hearing loss in this family was caused by novel compound heterozygous mutations in *TMC1*.

## Introduction

Hearing loss is a common sensory defect that can significantly impact quality of life, and the majority of congenital cases of hearing loss are attributable to genetic factors [1]. Non-syndromic hereditary forms, in which the hearing loss is the only clinical sign, are genetically heterogeneous. Autosomal recessive nonsyndromic hearing loss (ARNSHL) is the most common type and accounts for ∼80% of cases of inherited hearing loss. To date, 61 genes and more than 100 genetic loci have been implicated in ARNSHL (http://hereditaryhearingloss.org/). The marked heterogeneity of genetic hearing loss can be explained by the complexity of the auditory system, which requires coordination of multiple processes involving the inner ear and nervous system. A defect in any part of this complex chain of events can lead to hearing impairment. However, despite previous intensive linkage analysis and candidate gene screening, a large proportion of ARNSHL remain genetically unexplained.

Next-generation sequencing, in particular whole-exome sequencing (WES) involving the targeted sequencing of the protein-coding subset of the human genome, has become a highly efficient strategy for identifying novel causative genes and mutations involved in heritable disease. Both simple nonsyndromic and complex syndromic forms of hearing loss can be resolved efficiently using WES, especially small families with distinct and interesting phenotypes that are too small to map [2], [3]. To date, nine syndromic or nonsyndromic deafness genes have been identified using targeted genomic enrichment and next-generation sequencing including *TPRN*, *GPSM2*, *CEACAM16*, *SMPX*, *HSD17B4*, *HARS2*, *MASP1*, *DNMT1*, *TSPEAR* [4], [5], [6], [7], [8], [9], [10], [11], [12].

TMC1 is predicted to be involved in the functional maturation of cochlear hair cells [13]. The precise function of the TMC1 protein in the inner ear is unknown although its expression is localized to outer hair cells of the mouse inner ear after P3 [14]. Mutations in this gene at the DFNA36 and DFNB7/11 loci have previously been reported to cause nonsyndromic autosomal dominant and recessive hearing loss, respectively [15]. *TMC1* mutations are the sixth most common causes of ARNSHL in populations from North Africa, the Middle East, and India [16], [17], [18], [19]. To date, 35 recessive mutations in *TMC1* were shown to be associated with ARNSHL at the DFNB7/11 locus in more than 40 families worldwide (Table 1). However, these mutations were not found in Chinese populations. Almost all reported recessive cases showed a similar phenotype characterized by prelingual severe-to-profound hearing loss.

**Table 1:** Overview of all *TMC1* mutations identified to date.

Origin	MutationDNA	Protein	Exon(E)/Intron(I)	Type ofvariant	Inheritance pattern	Reference
North Ameirica	c. 1714 G>A	p. D572N	E19	Missense	AD	[15], [24], [29]
North Ameirica	c. 1714 G>C	p. D572H	E19	Missense	AD	[30]
China	c.589G>A	p.G197R	E11	Missense	AR	Present study
China	c.1171C>T	p.Q391X	E15	Nonsense	AR	Present study
PakistanTunisia Lebanon JordanTurkey	c. 100 C>T	p. R34X	E7	Nonsense	AR	[15], [19], [24], [30], [31]
Pakistan	c. IVS3_IVS5	n.d.	E5	Deletion	AR	[15]
PakistanIndia	c. 295_296 delA	n.d.	E8	Deletion	AR	[15]
Pakistan	c. IVS10-8T>A	n.d.	I10	Splice site mutation	AR	[15]
Pakistan	c. IVS13+1G>A	n.d.	I13	Splice site mutation	AR	[15], [32]
Turkey	c. 776 A>G	p. Y259C	E13	Missense	AR	[25]
Turkey	c.821C>T	p.P274L	E13	Missense	AR	[25]
Turkey	c. 1083_1087delCAGAT	p.R362P*fs*X6	E15	Deletion	AR	[25]
TurkeyPakistan	c. 1334 G>A	p. R455H	E16	Missense	AR	[25], [32]
Pakistan	c. 1534C>T	p. R512X	E17	Nonsense	AR	[15]
India	c.1960A>G	p.M654V	E20	Missense	AR	[15]
Pakistan	c.884+1G>A		E13	Splice site mutation	AR	[15]
Pakistan	c.830A>G	p.Y277C	E13	Missense	AR	[32]
Pakistan	c.536-8T>A		E11	Splice site mutation	AR	[32]
Pakistan	c.1114G>A	p.V372M	E15	Missense	AR	[32]
Pakistan	c.2004T>G	p.S668R	E21	Missense	AR	[32]
Pakistan	c.2035G>A	p.E679K	E21	Missense	AR	[32]
SudanTunisiaLebanonJordan	c.1165C>T	p.R389X	E15	Nonsense	AR	[18], [24], [31]
Sudan	c.IVS19+5G>A	n.d.	I19	Splice site mutation	AR	[18]
Pakistan	c. 1541C>T	p.P514L	E17	Missense	AR	[17]
Pakistan	c.1543T>C	p.C515R	E17	Missense	AR	[17]
Pakistan	c.IVS5+1G>T	Splice Site	I5	Splice site mutation	AR	[17]
Greece	c.2350C>T	p.R604X	E20	Nonsense	AR	[24]
Iran	c.776+1G>A	Splice Site	E7	Splice site mutation	AR	[24]
Turkey	c.767delT	p.F255FfsX14	E13	Deletion	AR	[24]
Turkey	c.1166G>A	p.R389Q	E15	Missense	AR	[24]
Tunisia	c.1764G>A	p.W588X	E19	Nonsense	AR	[31]
Turkey	c.1330G>A	p.G444R	E16	Missense	AR	[19]
Turkey	c.1333C>T	p.G445R	E16	Missense	AR	[19]
Turkey	c.2030T>C	p.I667T	E21	Missense	AR	[19]
Turkey	c.IVS6+2T>A	Splice Site	I6	Splice site mutation	AR	[19]
Turkey	c.1685_2280 del		E19–24	Deletion	AR	[19]
The Netherlands	c.1763+3A>G	p.W588WfsX81	I19	Splice site mutation	AR	[16]

AD, Autosomal Dominant; AR, Autosomal Recessive.

Here, we report a family with two siblings affected by sensorineural hearing loss. Mutations in *GJB2* and *SLC26A4* were excluded previously. As the family is small and non-consanguineous, neither linkage analysis nor homozygosity mapping would have been informative in identifying the causative gene. Therefore, we used WES to identify the gene responsible for the disease in this family. WES was carried out in two affected siblings, followed by validation in the family. The results identified two compound heterozygous disease-segregating mutations, c.589G>A (p.G197R) and c.1171C>T (p.Q391X), in the *TMC1* gene. To exclude the possibility that these mutations were polymorphisms, DNA samples of 50 affected and 208 unaffected individuals were also analyzed.

## Materials and Methods

### Clinical Data

Family 1953 is a two-generation Chinese family with autosomal recessive prelingual non-syndromic sensorineural hearing loss. To screen for candidate mutations, we used 208 ethnicity-matched controls and 50 affected DNA samples from the Department of Otolaryngology, Head and Neck Surgery, Chinese PLA General Hospital. The fifty affected individuals were from families presenting with ARNSHL and in whom mutations of *GJB2* and *SLC26A4* had been excluded previously. Fully informed written consent was attained from each subject or their guardians. The study was approved by the Chinese PLA General Hospital Research Ethics Committee. Medical histories of the members of family 1953 were obtained using a questionnaire regarding the following aspects of this condition: subjective degree of hearing loss, age at onset, progression, symmetry of the hearing impairment, use of hearing aids, presence of tinnitus, medication, noise exposure, pathological changes in the ear, and other relevant clinical manifestations. Otoscopy, physical examination, and pure tone audiometric examination (at frequencies from 250 to 8000 Hz) were performed to identify the phenotype. Immittance testing was applied to evaluate middle-ear pressure, ear canal volumes, and tympanic membrane mobility. Unaffected phenotype status was defined by threshold lower than age- and gender-matched 50^th^ percentile values for all frequencies measured. Computed tomography (CT) scan of the temporal bone was performed. The diagnosis of profound sensorineural hearing impairment was made according to the ICD-10 criteria based on audiometric examination. Tandem gait and Romberg tests were performed to evaluate balance.

All genomic DNA was extracted from peripheral blood using a blood DNA extraction kit according to the protocol provided by the manufacturer (TianGen, Beijing, China).

### Targeted Gene Capture and Sequencing

Approximately 99.71% of CCDS exons or 99.62% of RefSeq exons from 6 µg of genomic DNA were captured using the NimblegenSeqCap EZ Library (44 Mb for II:1 and II:2). Genomic DNA sample was randomly fragmented by Covaris; the size of the library fragments was distributed mainly between 250 and 300 bp. Then, adapters were ligated to both ends of the resulting fragments. Extracted DNA was then amplified by ligation-mediated PCR (LM-PCR), purified, and hybridized to the NimblegenSeqCap EZ Library for enrichment; non-hybridized fragments were then washed out. Both non-captured and captured LM-PCR products were subjected to quantitative PCR to estimate the magnitude of enrichment. Each captured library was then loaded onto the Illumina Hiseq2000 platform, and we performed high-throughput sequencing for each captured library to ensure that each sample met the desired average sequencing depth. Raw image files were processed by Illumina base calling Software 1.7 for base calling with default parameters and the sequences of each individual were generated as 90-bp pair-end reads.

### Reads, Mapping, and Variant Detection

SNP detection was performed as follows: (i) SOAPaligner (version 2.21) [20] was used to align the high-quality reads to the human reference genome (hg19, NCBI build 37.1); (ii) for paired-end reads with duplicated start and end sites, only one copy with the highest quality was retained, and the reads with adapters were removed; (iii) SOAPsnp (version 1.05) [21] was used to assemble the consensus sequence and call genotypes. For SNP quality control, low-quality SNPs that met one of the four following criteria were filtered out: (i) genotype quality less than 20; (ii) sequencing depth of less than 4 for the site; (iii) estimated copy number more than 2; (iv) distance from the adjacent SNPs less than 5 bp. Small indel detection was performed using the UnifiedGenotyper tool from GATK (version v1.0.4705) [22] after all the high-quality reads were aligned to the human reference genome using BWA (version 0.5.9-r16) [23]. SNP and indel detection were performed only on the targeted exome regions and flanking regions within 200 bp.

### Filtering and Annotation

The detected variants were annotated and filtered based on four databases; *i.e.*, NCBI CCDS (http://www.ncbi.nlm.nih.gov/CCDS/CcdsBrowse.cgi), RefSeq (http://www.ncbi.nlm. nih.gov/RefSeq/), Ensembl (http://www.ensembl.org), and Encode (http://genome.ucsc.edu/ENCODE). Four major steps were taken to prioritize all the high-quality variants: (i) variants within intergenic, intronic, and UTR regions and synonymous mutations were excluded from downstream analysis; (ii) variants in dbSNP132 (http://www.ncbi.nlm.nih.gov/projects/SNP/), 1000 Genome project (ftp://ftp.1000genomes.ebi.ac.uk/vol1/ftp), YH Database (http://yh. genomics.org.cn/), and HapMap Project (ftp://ftp.ncbi.nlm.nih.gov/hapmap) were excluded; (iii) Possible impacts of variants were predicted by SIFT (http://sift.bii. a-star.edu.sg/) and Polyphen2 (http://genetics.bwh.harvard.edu/pph2/); (iv) Gene Ontology (http://www. geneontology.org) and KEGG Pathway Annotations (http://www.ebi.ac.uk./clustalw) were used.

### Mutation Validation

After filtering against multiple databases, Sanger sequencing was used to determine if any of the potential novel mutations in known causative genes of ARNSHL co-segregated with the disease phenotype in this family. Direct polymerase chain reaction (PCR) products were sequenced using Bigdye terminator v3.1 cycle sequencing kits (Applied Biosystems, Foster City, CA) and analyzed using an ABI 3700XL Genetic Analyzer.

### Mutational Analysis

Segregation of the mutations was studied in all family members. In addition, 208 negative samples and 50 ARNSHL families were also screened for the mutations by direct sequencing. Genotyping for c.589G>A and c.1171C>T was performed by PCR (primer sequences are available on request) and detected by bidirectional sequencing of the amplified fragments using an automated DNA sequencer (ABI 3100, Applied Biosystems). Nucleotide alteration(s) were identified by sequence alignment with the *TMC1*GenBank sequence using Genetool software.

### Multiple Sequence Alignment

Multiple sequence alignment was performed using Homologene with the default settings and the sequences NP_619636.2 (*H. sapiens*), XP_001093188.2 (*M. mulatta*), XP_528322.2 (*P. troglodytes*), XP_002689695.1 (*B. taurus*), XP_541284.3 (*C. lupus*), NP_083229.1 (*M. musculus*), NP_001101991.1 (*R. norvegicus*), NP_001006580.1 (*G. gallus*), and XP_695621.3 (*D. rerio*). (http://www.ncbi.nlm.nih.gov/homologene?cmd=Retrieve&dopt=MultipleAlignment&list_uids=23670).

## Results

### Clinical Presentation of Family 1953

**Figure 1:**
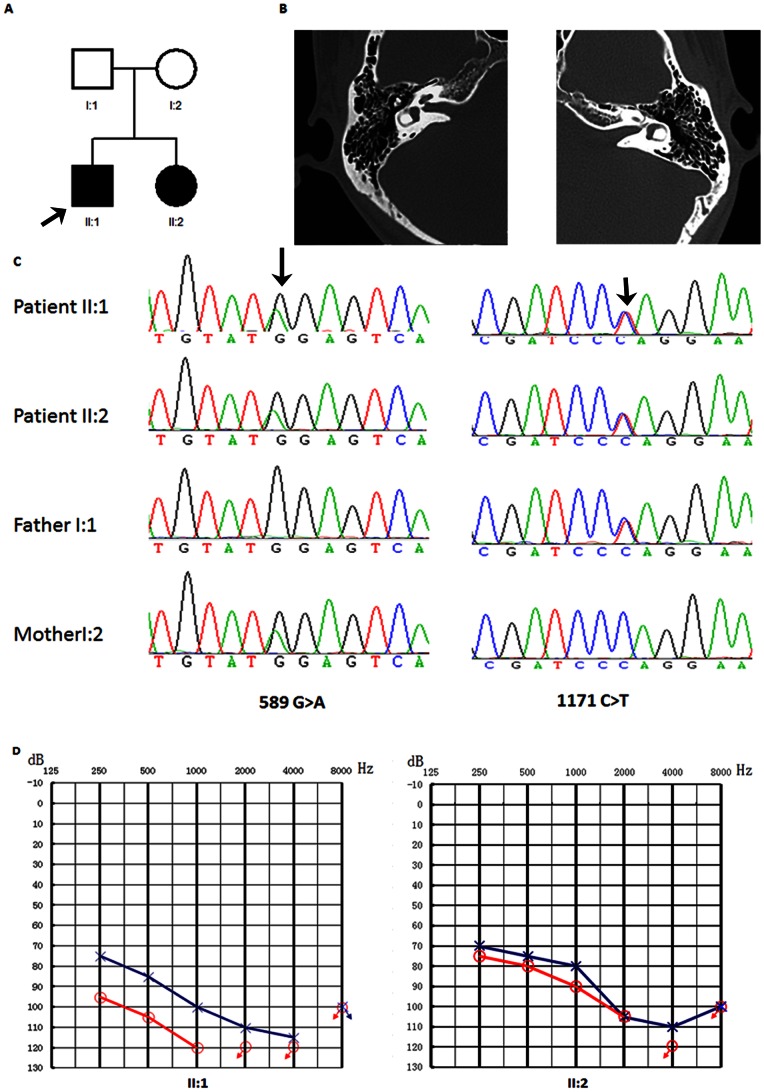
Combined figure. A. Pedigree of Family 1953 with ARNSHL Affected subjects are denoted in black. Arrow indicates the proband; B. temporal bone CT of the II:1 shows no structural change; C. Electropherograms analysis of TMC1 in family 1953 showing the compound heterozygous mutations (c.589G>A and c.1171C>T) co-segregated with the phenotype. D. Audiogram of affected subjects showed hearing loss ranged from severe to profound.

We analyzed a two-generation ARNSHL family (family 1953) including two affected siblings (16 and 24 years old) and unaffected parents (Figure 1A). Audiograms of the two affected siblings showed that the hearing loss was bilateral, severe to profound, with some residual hearing at low frequency (Figure 1D). The stable nature of hearing loss was reported by the affected individuals but was not verified with audiograms. Immittance testing demonstrated normal and bone conduction values equal to the air conduction measurements, suggesting a sensorineural hearing impairment. Affected individuals did not have delays in gross motor development; neither did they have balance problems, vertigo, dizziness, nor spontaneous and positional nystagmus. Tandem walking was normal and Romberg test was negative. CT scan of the temporal bone in the proband excluded inner-ear malformations (Figure 1B). The parents reported no history of stillbirth or miscarriage. Physical examination of all members revealed no signs of systemic illness or dysmorphic features. The remaining examination results were completely normal. Affected individuals did not have delayed gross motor development. This phenotype is consistent with that reported previously for DFNB7/11 [15], [19], [24]. To exclude mutations in the genes commonly known to be associated with hereditary hearing loss, *SLC26A4* and *GJB2* were sequenced in the proband (patient II:1 in Figure 1A). No mutations were found in these genes and we proceeded to sequence the whole exomes of the two patients.

### Whole-exome Sequencing

By WES of the proband II:1 and proband II:2, we generated 6.4 and 5.9 billion bases of sequence with mean target region depths of 61.93 and 66.68, respectively. Approximately 99% and 98.98% (2739 Mb and 2942 Mb in length, respectively) of the targeted bases were covered sufficiently to pass our thresholds for calling SNPs and short insertions or deletions (indels). The rates of nucleotide mismatch were 0.37% and 0.36%, respectively. After identification of variants, we focused only on non-synonymous variants (NS), splice acceptor and donor site mutations (SS), and frameshift coding insertions of deletions (indels), which were more likely to be pathogenic than others, especially those in homozygous or multiple heterozygous mode.

In II:1 and II:2, we separately identified 12192 and 12395 SNPs in the coding regions (11975 and 12171 missense, 72 and 74 readthrough, 145 and 150 nonsense), 2527 and 2557 variants in introns that may affect splicing (within 10 bp of the intron/exon junction), respectively. Then we compared these variants in two affected members with the dbSNP135, HapMap project, 1000 Genome Project, and YH database. Under the autosomal recessive model, we identified 113 genes with homozygous or multiple heterozygous variants (216 SNPs and 59 indels) common to both affected siblings. Then, we compared these variants with reported Nonsyndromic Hereditary Hearing Loss genes (http://hereditaryhearingloss. org/), in which six mutations were found in previously reported deafness-related genes (Table 2).

**Table 2:** Candidate SNP calls identified in 2 affected siblings.

Chr:Position	Gene	Mutation	Mode	Mutation type	Maternal allele	Paternal allele	SIFT/Polyphen2	Expressed in inner ear	Deafness gene	frequencies in the Exome variant Server
1∶16354325	CLCNKA	791c>a	heterozygous	missense		Yes	Damaging/Benign	Yes	N/A	N/A
1∶16360198	CLCNKA		heterozygous	5′-UTR	Yes		N/A	Yes	N/A	N/A
16∶21747633	OTOA	2353a>c	heterozygous	missense	Yes		Tolerated/Benign	Yes	Yes	N/A
16∶21747639	OTOA	2359g>t	heterozygous	nonsense	Yes		Damaging/possibly damaging	Yes	Yes	N/A
9∶75366819	TMC1	589g>a	heterozygous	missense	Yes		Damaging/possibly damaging	Yes	Yes	N/A
9∶75404180	TMC1	1171c>t	heterozygous	nonsense		Yes	Damaging/possibly damaging	Yes	Yes	N/A

### Mutation Detection and Analysis

SIFT and Polyphen2 were used as a first-pass filter to predict how the identified amino acid substitutions would affect protein function taking into account sequence homology and the physical properties of amino acids. Two novel mutations in the previously described pathogenic gene *TMC1* were predicted to be damaging: *TMC1* c.589G>A (G197R) and *TMC1* c.1171G>A (Q391X) (according to GenBank accession number NM_138691.2). The mutation c.589G>A located within exon 11 and predicted to lie within hydrophobic transmembrane domains changes a hydrophilic glycine to a hydrophobic alanine at position 197, and may lead to a change in protein tertiary structure. Mutation c.1171G>A located within exon 15 results in the nonsense mutation Q391X, and the premature stop codon is likely to activate the nonsense-mediated mRNA decay response, thus leading to a decrease in TMC1 mRNA expression.

**Figure 2:**
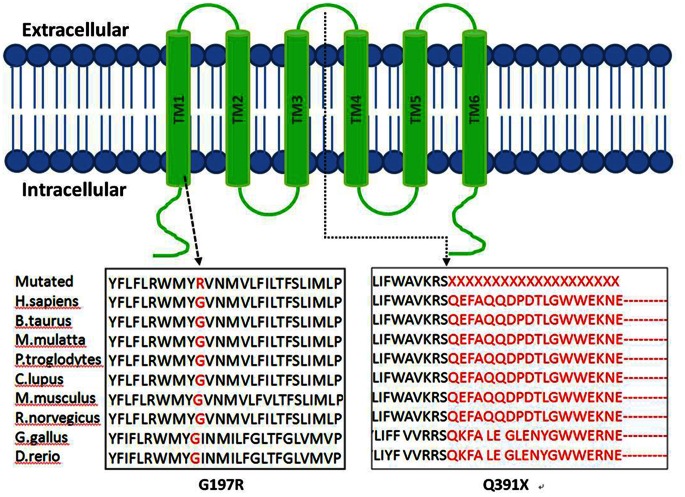
Schematic structure of TMC1 and Conservation analysis. G197R occur in the second TMhelix, Q391X occur extracellular between TM3 and TM4. Protein alignment showed conservation of residues TMC1 G197 and Q391 across nine species. These two mutations occur at an evolutionarily conserved amino acid (in red).

Both amino acid changes affect highly preserved residues (Figure 2). Sanger sequencing validated the two affected siblings’ mutations and demonstrated that the parents were unaffected carriers of G197R (mother) and Q391X (father) deletion, showing complete co-segregation of the mutations with the phenotype (Figure 1C).

By direct sequencing, 208 ethnicity-matched negative controls and 50 Chinese familial patients with ARNSHL were genotyped to screen for the mutations. The two mutations described above were absent in 208 negative samples and in 50 ARNSHL families.

These data, together with the clinical presentation of the two affected siblings, indicate clearly that *TMC1* mutations were responsible for ARNSHL in this family.

We used TMHMM2.0 to predict the TMC1 protein with six membrane-spanning regions and cytoplasmic N- and C-termini. The result indicated that G197R occurs in the second TM helix, while Q391X occurs in the extracellular region between TM3 and TM4 (Figure 2). This predicted model of TMC1 membrane topology was similar to those of ion channels or transporters.

## Discussion

For many decades, linkage analysis has been the most powerful and widely used strategy to identify the gene defects responsible for inherited disorders. However, this approach is time consuming, and requires the availability of cohorts of homogeneous and informative families. Homozygosity mapping is a potentiated version of linkage analysis, which makes possible the identification of several dozens genes responsible for autosomal recessive diseases, but it requires the availability of informative, possibly large, consanguineous families. As the molecular basis of deafness in most of our Chinese probands was unsolved, we predict that many new hearing loss genes and mutations remain to be identified. Next-generation sequencing can now be performed rapidly and at minimal cost, allowing analysis of the coding regions (exome) of the human genome in single individuals or small families, including patients in whom a clear genotype-phenotype correlation is absent or for clinically and genetically heterogeneous conditions. WES provides unprecedented opportunities to identify causative DNA mutations in rare heritable disorders.

This represents the first report of novel compound heterozygous mutations in the *TMC1* gene as a cause of ARNSHL. The *TMC1* gene on chromosome 9q12 contains 24 exons, including four exons encoding sequence upstream of a methionine codon in exon 5, and normally encodes a product of 760 amino acids. *TMC1* mutations seem to be rather common causes of recessive deafness in India, Pakistani, Turkish, and Tunisian families [15], [19], [25].

*TMC1* is predicted to contain six transmembrane domains, and both C- and N-termini are located intracellularly; this protein exhibits no apparent amino acid sequence similarity to any other proteins of known function. Expression analysis of *TMC1* resulted in detection of transcripts in human fetal cochlea and mouse inner and outer cochlear hair cells as well as in neurosensory epithelia of the vestibular organs [15], and is required for postnatal hair cell development and maintenance [14].

*Tmc1* deficiency in the recessive deafness mouse mutant *dn* leads to complete deafness and hair cell degeneration, suggesting that *Tmc1* is necessary for maturation and survival of hair cells in the murine cochlea.

Mouse models with the *Tmc1* defect [14], [15] support a role for Tmc1 in the inner and outer hair cells, either in proper trafficking of other membrane proteins in these cells or in regulating the differentiation of immature hair cells into fully functional auditory receptors [13]. This model suggested that mechanical forces brought about by bending of stereocilia and tension on the tip links directly activate ion channels. If TMC1 is an ion channel that is mainly localized in the IHC, then it may be involved in the most basic auditory process of hair cell transduction.

The predicted structure of TMC1 bears similarity to that of the α-subunit of voltage-dependent K^+^ channels, which have six α-helical TM segments and intracellular N- and C-termini [26]. TMC1 has been predicted to be an ion channel or transporter that mediates K^+^ homeostasis in the inner ear [27]. Ion channels serve many functions, including the transport of ions and water, the control of electrical excitability, and the regulation of ionic homeostasis. The first four TM domains of the K^+^ channel α-subunit act as voltage sensors for activation gating [28], whereas the intervening segment between TM5 and TM6 appears to confer channel selectivity [26]. *TMC1* c.589G>A (G197R) lies in TM2 and c.1171G>A (Q391X) lies between TM3 and TM4 extracellularly, and the mutations may affect an ion channel function in hair cells.

The stop codon in exon 15 (c.1171G>A [Q391X]) identified in this study is close to the previously reported mutation c.1165G>A [Q389X], and is predicted to result in formation of a truncated protein with impaired function [18].

In summary, we have reported the clinical and genetic characteristics of a non-consanguineous Chinese family with ARNSHL by WES. A major challenge for mutation discovery by WES is determining which variants are potentially causative and which are likely to be benign. To identify pathogenic variants, we consecutively filtered these variants by subjecting them to an analytical pipeline for high-confidence variant calling and annotation and identified novel compound heterozygous mutations in *TMC1.* The identification of additional mutations in *TMC1* further confirms its crucial role in auditory function. These results support sequence analysis of *TMC1* in clinical diagnostic testing of individuals with ARNSHL, as families with an affected child both parents of which are carriers have a 25% risk of recurrence. Moreover, pre-implantation genetic diagnosis is possible.

The English in this document has been checked by at least two professional editors, both native speakers of English. For a certificate, please see: http://www.textcheck.com/certificate/KuK2Ub.
